# Multivariable clinical-genetic risk model for predicting venous thromboembolic events in patients with cancer

**DOI:** 10.1038/s41416-018-0027-8

**Published:** 2018-03-28

**Authors:** Andrés J. Muñoz Martín, Israel Ortega, Carme Font, Vanesa Pachón, Victoria Castellón, Virginia Martínez-Marín, Mercedes Salgado, Eva Martínez, Julia Calzas, Ana Rupérez, Juan C. Souto, Miguel Martín, Eduardo Salas, Jose M. Soria

**Affiliations:** 1 Medical Oncology Service, Hospital General Universitario Gregorio Marañón, C/ Doctor Esquerdo, 46, 28007 Madrid, Spain; 2 Cancer & Thrombosis Working Group, Spanish Society of Medical Oncology (SEOM), C/ de Velázquez, 7, 28001 Madrid, Spain; 3 Scientific Department, Gendiag, Joan XXIII, 10, 08950 Esplugues de Llobregat, Spain; 4 Medical Oncology, Hospital Clínic, c/ Villarroel, 170, 08036 Barcelona, Spain; 5 Medical Oncology, Hospital Universitario Ramón y Cajal Madrid, Ctra. Colmenar Viejo, Km. 9,100, 28034 Madrid, Spain; 6 Medical Oncology, Complejo Hospitalario de Torrecárdenas, c/ Hermandad de Donantes de Sangre, 04009 Almería, Spain; 7 Medical Oncology, Hospital Universitario la Paz, Paseo de la Castellana, 261, 28046 Madrid, Spain; 8 Medical Oncology, Complejo Hospitalario Universitario de Ourense, c/ Ramon Puga Noguerol, 54, 32005 Ourense, Spain; 9 Medical Oncology, Hospital Universitario Marqués de Valdecilla, Av. Valdecilla, 25, 39008 Santander, Spain; 10 Medical Oncology, Hospital Universitario de Fuenlabrada, Camino del Molino, 2, 28942 Madrid, Spain; 11 Medical Oncology, Fundación Jiménez Díaz, Av. Reyes Católicos, 2, 28040 Madrid, Spain; 12 Thrombosis and Haemostasia, Hospital de la Santa Creu i Sant Pau, Carrer de Sant Quintí, 89, 08041 Barcelona, Spain; 13 Genomic of complex diseases, Institut d’Investigació Sant Pau (IIB-Sant Pau), Carrer de Sant Quintí, 89, 08041 Barcelona, Spain

**Keywords:** Predictive markers, Genetic testing

## Abstract

**Background:**

Venous thromboembolism (VTE) is a leading cause of death among patients with cancer. Outpatients with cancer should be periodically assessed for VTE risk, for which the Khorana score is commonly recommended. However, it has been questioned whether this tool is sufficiently accurate at identifying patients who should receive thromboprophylaxis. The present work proposes a new index, TiC-Onco risk score to be calculated at the time of diagnosis of cancer, that examines patients’ clinical and genetic risk factors for thrombosis.

**Methods:**

We included 391 outpatients with a recent diagnosis of cancer and candidates for systemic outpatient chemotherapy. All were treated according to standard guidelines. The study population was monitored for 6 months, and VTEs were recorded. The Khorana and the TiC-Onco scores were calculated for each patient and their VTE predictive accuracy VTEs was compared.

**Results:**

We recorded 71 VTEs. The TiC-Onco risk score was significantly better at predicting VTE than the Khorana score (AUC 0.73 vs. 0.58, sensitivity 49 vs. 22%, specificity 81 vs. 82%, PPV 37 vs. 22%, and NPV 88 vs. 82%).

**Conclusions:**

TiC-Onco risk score performed significantly better than Khorana score at identifying cancer patients at high risk of VTE who would benefit from personalised thromboprophylaxis.

## Introduction

Patients with cancer (taking all types together) are at 7 times the risk of developing a venous thromboembolism (VTE); in some malignancies the risk increases to 28 times.^1^ The incidence of cancer-associated VTE is particularly high during the first few months after diagnosis, when distant metastases are present, and after initiating chemotherapy.^1, 2^ VTE is a leading cause of death among patients with cancer,^3^ and the survival of an episode may have clinical and economic implications, including hospitalisation, potential delays in cancer therapy, recurrent VTE, post-thrombotic syndrome, and chronic thromboembolic pulmonary hypertension. Indeed, these problems are common, costly, and have a profound impact on the patient's quality of life.^3^

Different guidelines cover the identification of patients with cancer at risk of VTE, VTE prevention strategies, and treatment.^4–6^ These documents indicate that most hospitalised patients with active cancer require thromboprophylaxis throughout hospitalisation. However, in the outpatient setting, it is indicated only for high-risk patients. Outpatients with cancer should be periodically assessed for VTE risk, for which the validated risk assessment tool developed by Khorana—the Khorana score^7^ is commonly recommended. However, in recent years, a number of studies have questioned whether this tool is sufficiently accurate at identifying patients who should receive thromboprophylaxis.^8–10^

In the present work, we hypothesised that a genetic component is involved in the appearance of VTE, a factor that the Khorana score does not take into account. Recently, the Vienna Group^11^ showed that the Leiden (rs6025) variant of the gene coding for factor V in the coagulation pathway doubles the risk of a VTE event occurring in patients with cancer. This variant might, therefore, provide a promising biomarker of venous thrombosis in such patients, and could be used in individual risk prediction.^12^ Other genes are known to increase the risk of VTE in the general population, and a tool (the Thrombo inCode or TiC tool) involving them as markers has been developed to predict VTE—but only for non-oncological populations.^13^ The use of such tools ought to allow more tailored thromboprophylaxis strategies to be followed.

The present work proposes a TiC-derived risk score—the TiC-Onco risk score—which takes into account both genetic and clinical risk factors, and which can be used to identify patients with cancer in the outpatient setting who are at high risk of VTE. Its capacity to identify such patient was compared with that of the Khorana score. The results suggest that the TiC-Onco risk score can better predict which patients should receive thromboprophylaxis.

## Patients and Methods

### Study design and participants

The study protocol was approved by the participant hospitals’ institutional review boards. Signed informed consent was obtained from each patient.

This study—the ONCOTHROMB12-01 study (Clinicaltrial.gov indentifier: NCT03114618)—is an observational cohort study involving an 18 month monitoring period with analysis at 6, 12, and 18 months. This paper presents the results for the first 6 months.

Selection criteria were as follows:

- Over 18 years of age
- Recent diagnosis of cancer of the following types: colorectal, oesophago-gastric, lung, or pancreatic.
- ECOG/WHO/Zubrod score of 0–2
- Candidates for systemic outpatient chemotherapy according to standard guidelines.
- No outpatient thromboprophylactic therapy deemed mandatory by the treating oncologist.

The Khorana score (reference tool) and the proposed TiC-Onco score (index tool) were calculated for each patient at the moment of initial diagnosis and their accuracy in terms of predicting the observed VTE events of the two tools was compared.

### Diagnosis of VTE events

Deep vein thrombosis in the lower limbs was diagnosed by ultrasound or ascending venography. Pulmonary embolism was diagnosed by ventilation–perfusion lung scanning, pulmonary angiography, or spiral computed tomography. Intracranial venous thrombosis was diagnosed by magnetic resonance imaging.

### Development of the TiC-Onco risk score

The TiC-Onco risk score tool was developed in three steps:

1. Development of a genetic risk score.

A total of 391 patients were genotyped for the genes shown in Table 1 using blood extracted at the time of diagnosis, employing TaqMan genotyping assays and the EP1 Fluidigm platform (an efficient endpoint PCR system for high-sample-throughput SNP genotyping). At 6 months, multivariate logistic regression analysis was performed to determine the weight of each genetic variable in the appearance of a VTE event. The final genetic risk score was determined using the genetic variants associated with an increased risk of VTE in the multivariate model (*p* ≤ 0.25).

**Table 1:** Study population characteristics

	VTE	No-VTE	*p*-value
N	71	320	
Sex (female), *n* (%)	27 (38.0)	108 (33.8)	0.584
Age, mean (sd)	64.1 (11.0)	64.3 (10.5)	0.903
Diabetes, *n* (%)	12 (16.9)	62 (19.4)	0.754
Smoking, *n* (%)	21 (29.6)	66 (20.6)	0.138
Family history (%)	6 (8.4)	12 (3.7)	0.112
BMI >25, *n* (%)	36 (50.7)	144 (45.0)	0.459
Hypercholesterolemia, *n* (%)	29 (40.8)	106 (33.1)	0.271
Hypertension (%)	33 (46.5)	141 (44.1)	0.932
Khorana ≥3	16 (22.5)	58 (18.1)	0.505
Primary site of tumour:			
Colon	22 (31.0)	141 (44.1)	0.059
Pancreas	29 (40.8)	43 (13.4)	<0.001
Lung	11 (15.5)	76 (23.8)	0.175
Oesophagus	2 (2.8)	12 (3.7)	0.976
Stomach	7 (9.9)	48 (15.0)	0.348
Tumour Stage:			
I + II	5 (7.0)	66 (20.6)	0.012
III	18 (25.4)	121 (37.8)	0.065
IV	48 (67.6)	133 (41.6)	<0.001
Haemoglobin <100 g/L, n (%)	4 (5.6)	18 (5.6)	>0.999
Platelet >350 × 10^9^/L, n (%)	13 (18.3)	74 (23.1)	0.469
Leukocyte >11 × 10^9^/L	15 (21.1)	58 (18.1)	0.675
SNPs, risk alleles (%)			
F5 rs6025			
0 Risk Alleles	68 (95.8)	314 (98.1)	0.213
1 Risk Allele	3 (4.2)	6 (1.9)	
F5 rs4524			
0 Risk Alleles	1 (1.4)	22 (6.9)	0.108
1 Risk Allele	22 (31.0)	115 (35.9)	
2 Risk Alleles	48 (67.6)	183 (57.2)	
F2 rs1799963			
0 Risk Alleles	69 (97.2)	307 (95.9)	>0.999
1 Risk Allele	2 (2.8)	12 (3.7)	
2 Risk Alleles	0	1 (0.3)	
F12 rs1801020			
0 Risk Alleles	46 (64.8)	204 (63.7)	>0.999
1 Risk Allele	23 (32.4)	103 (32.2)	
2 Risk Alleles	2 (2.8)	13 (4.1)	
F13 rs5985			
0 Risk Alleles	36 (50.7)	184 (57.5)	0.514
1 Risk Allele	30 (42.3)	119 (37.2)	
2 Risk Alleles	5 (7.0)	17 (5.3)	
SERPINC1 rs121909548			
0 Risk Alleles	71 (100.0)	319 (99.7)	>0.999
1 Risk Allele	0	1 (0.3)	
SERPINA10 rs2232698			
0 Risk Alleles	68 (95.8)	314 (98.1)	0.213
1 Risk Allele	3 (4.2)	6 (1.9)	
A1 blood group			
0 A1 Allele	41 (57.7)	194 (60.6)	0.776
1 A1 Allele	24 (33.8)	105 (32.8)	
2 A1 Alleles	6 (8.4)	21 (6.6)	

2. Selection of clinical variables associated with the development of VTE.

Data were collected from all patients on the clinical risk factors cited in the literature^14^ as being associated with VTE and that could be known at the time of diagnosis: primary tumour site, tumour node metastasis stage, and body mass index (BMI), use of tobacco, age, sex, family (first degree) history of VTE, the presence of diabetes, hypertension, and high blood cholesterol level, the Khorana score, previous surgery, number of platelets, number of leukocytes, and immobilisation. The risk of VTE associated with the primary tumour site (low, high, and very high) was categorised as when determining the Khorana score.^15^ The risks associated with platelet and leukocyte numbers were categorised using the same cut-offs as for the Khorana score.^15^

 At 6 months, univariate analysis was performed to determine which of these variables were associated with the appearance of a VTE event. Those associated with an increased risk of VTE (*p* ≤ 0.25) were selected.

3. Development of the clinical-genetic model.

The genetic risk score and the clinical variables selected were subjected to multivariate logistic regression analysis using an AIC-based backward selection process.^16^

### Internal validation

Internal validation to obtain the degree of optimism in the area under the receiver operating characteristic (ROC) curve (AUC) estimation was done using the bootstrap approach,^17^ considering 100 resamples from the original data.

### Comparing the Khorana and TiC-Onco risk scores

The risk prediction capacity of the Khorana and TiC-Onco risk scores was evaluated using the area under the receiver operating characteristic (ROC) curve (AUC, larger values indicate better discrimination).^18^ Standard measures of sensitivity, specificity, positive, and negative predictive value (PPV and NPV), and positive and negative likelihood ratios (PLR and NLR),^19^ were determined for specific cut-off points.

For Khorana score the cut-off defining high risk was set at ≥3 (the normal cut-off value), and 0 for the low risk category definition. We contemplate two scenarios when determining a cut-off for the TiC-Onco score. In the first one (the main scenario), the cut-off is selected as the point on the ROC curve giving the same specificity as provided by the Khorana score (around 80%). It defines those individuals who are in high risk. This will be the default cut-off for TiC-Onco when not specified in the text. In this scenario, we also determine a second cut-off to classify the non-high risk individuals into either intermediate or low risk. Thus, we allow the TiC-Onco score to provide three risk categories—high, intermediate, and low risk. This second cut-off to discriminate between low and intermediate categories is selected as the point giving a sensitivity of 90%. The second scenario is presented just for informative purposes. In this case, the cut-off is selected as the point which maximises the Youden’s index (defined as sensitivity + specificity−1).^20^ In this scenario, we only consider a TiC-Onco with two categories, high risk and non-high risk.

### Statistical analysis

Continuous variables were recorded as median [1st–3rd Quartiles], and categorical variables as proportions. Univariate association between clinical/genetic variables and events was determined using either *t*-test or Wilcoxon rank sum tests for continuous variables, and χ^2^ or Fisher tests for categorical variables. All calculations were performed using R statistical software (version 3.1.3).^21^

### Number of patients needed to treat

To assess the effect of the TiC-Onco risk score in terms of preventing VTE events, the number of patients needed to treat (NNT) was determined for both scores.^22^ It was assumed that prophylactic medication would reduce cancer-associated VTE by 46%.^23^

## Results

### Patient characteristics

Tables 1 and 2 show the clinical and demographic characteristics of the 391 patients at the start of the study. For each variable, the number and percentage of patients who experienced a VTE, or not, at some point in the 6-month study period, are shown. The overall incidence of VTE was 18%. Patients suffering from pancreatic cancer experienced VTE at a significantly higher frequency (40%) than patients with other type of cancers (*p* < 0.001) (Table 2).

**Table 2:** Population charactheristics per cancer type

Tumour type	Colon	Pancreas	Lung	Oesophagus	Stomach	Total
Total	163	72	87	14	55	391
Patients (%)	41.69	18.41	22.25	3.58	14.07	100
No-VTE	141	43	76	12	48	320
VTE	22	29	11	2	7	71
% VTE	13.50	40.28	12.64	14.29	12.73	18.16
Stage I + II	29	18	9	3	12	71
Stage III	74	12	34	5	14	139
Stage IV	60	42	44	6	29	181
Death, *n* (%)	13 (7.98)	24 (33.33)	13 (14.94)	1 (7.14)	7 (12.73)	
VTE death, *n* (%)	5 (38.46)	14 (58.33)	3 (23.08)	0	0	
No-VTE deaths, *n* (%)	8 (61.54)	10 (41.67)	10 (76.92)	1 (100)	7 (100)	

### Development of the TiC-Onco risk model

Table 3 shows the genetic and clinical markers that were significantly associated by multivariate analysis with a VTE event, and thus selected for inclusion in the TiC-Onco risk score model.

**Table 3:** Clinical-genetic risk score Thrombo inCode-Oncology (TiC-Onco)

Variable	*p*-value
GRS	0.0049
BMI >25	0.0658
Family history	0.1076
Primary tumour site	
HR	0.3483
VHR	0.0033
Tumour stage	0.0003
GRS	
rs2232698	0.1460
rs6025	0.2064
rs5985	0.2003
rs4524	0.0396

*GRS* Genetic Risk Score, *HR* High Risk, *VHR* Very High Risk

### Accuracy and validation of the risk model

The TiC-Onco score showed an AUC of 0.73 (0.67–0.79), a sensitivity of 49%, and a specificity of 81%. Its PPV was 37%, NPV 88%, PLR 2.6, and NLR 0.6% (Table 4). The Khorana score showed a significantly lower capacity to distinguish between patients who experienced/did not experience a VTE event (AUC 0.73 vs. 0.58; *p* < 0·001). The sensitivity of the TiC-Onco score was significantly higher than that of the Khorana (49 vs. 22%; *p* < 0.001), while the specificities of both scores were similar (81 vs. 82%; *p* = 0.823). The PPV and NPV of the TiC-Onco score were significantly higher than those of the Khorana score (37 vs. 22%; *p* = 0.004 for PPV and 88 vs. 82%; *p* < 0.001 for NPV). The LRs of the TiC-Onco score were also significantly better (Table 4).

**Table 4:** Predictive capability of TiC-Onco and Khorana scores

	TiC-Onco (1)	TiC-Onco (2)	Khorana	*p* (TiC-Onco (1) vs Khorana)	*p* (TiC-Onco (2) vs Khorana)
AUC (95% CI)	0.734 (0.67–0.79)	0.734 (0.67–0.79)	0.580 (0.51–0.65)	<0.001	<0.001
Sensitivity, % (95% CI)	49.30 (37.7–60.9)	85.92 (77.8–94.0)	22.54 (12.8–32-3)	<0.001	<0.001
Specificity, % (95%, CI)	81.25 (77.0–85.5)	49.06 (43.6–54.5)	81.76 (77.5–86.0)	0.823	<0.001
PPV, % (95% CI)	36.84 (27.1–46.5)	27.23 (21.4–33.1)	21.62 (12.2–31.0)	0.004	0.218
NPV, % (95% CI)	87.84 (84.1–91.6)	94.01 (90.4–97.6)	82.54 (78.3–86.7)	<0.001	<0.001
PLR (95% CI)	2.63 (1.89 - 3.65)	1.69 (1.46 - 1.95)	1.24 (0.76 - 2.02)	0.005	0.244
NLR (95% CI)	0.62 (0.49 - 0.79)	0.29 (0.16 - 0.52)	0.95 (0.83 - 1.09)	0.001	<0.001

TiC-Onco (1) shows the predictive capabilities for the default cut-off (see Methods). TiC-Onco (2) shows the predictive capabilities for the cut-off providing the best Youden’s Index.

*AUC* Area Under the Roc Curve, *PPV* Positive Predictive Value, *NPV* Negative Predictive Value, *PLR* Positive Likelihood Ratio, *NLR* Negative Likelihood Ratio

Table 5 shows the distribution of patients with or without VTE according to the Khorana score. The great majority of patients who suffered a VTE event (77%) were identified by the Khorana score as being at low or moderate risk (values 0, 1, and 2). Among these 55 patients, however, 17 (31%) were detected as high-risk patients by the TiC-Onco score. When the cut-off for high risk was taken as the best Youden Index, the TiC-Onco score returned significantly better predictions of risk than the Khorana score, especially in terms of sensitivity (86 vs. 22%, *p *< 0.001) (Table 4). In this scenario, of the 55 patients who experienced a VTE event (but who were classified as not being at high-risk by the Khorana score), 40 (73%) were detected as high risk patients by the TiC-Onco score. Table 6 shows rates of VTE according to prespecified risk categories for both TiC-Onco and Khorana.

**Table 5:** Patient distribution according to Khorana score considering patients with and without VTE

Khorana	VTE	No-VTE	Patients (*n*)	Patients (%)	% VTE
0	14	94	108	27.62	12.96
1	11	74	85	21.74	12.94
2	30	92	122	31.20	24.59
≥3	16	58	74	18.93	21.62
NA	0	2	2	0.51	0
Total	71	320	391		

Patients (%) percentage of patients per Khorana score level.

%VTE cases percentage of patients with VTE in relation to the number of patients per Khorana score level

**Table 6:** Percentage of study population among risk categories, and percentage of patients with VTE. See Methods for details about the definition of two and three categories

	TiC-Onco		Khorana	
	% of cancer population	% of cancer patient with VTE	% of cancer population	% of cancer patient with VTE
High risk	24.30	36.84	18.93	21.62
Non-high risk	75.70	12.16	—	—
Moderate risk	39.13	18.30	52.94	19.81
Low risk	36.57	5.59	27.62	12.96

The NNT values for: (a) if all patients included in the study had been treated (NNT = 12); (b) if only the patients with a Khorana score of ≥3 had been treated (NNT = 10), or (c) if only patients with a high risk TiC-Onco score (with the cut-off set at the same specificity as the Khorana score) had been treated (NNT = 6).

## Discussion

When deciding whether to use primary antithrombotic prophylaxis in outpatients with cancer who are candidates for chemotherapy, a clinician needs to determine the risk of VTE and weigh the likely benefit against the risk of bleeding. Despite the awareness of scientific societies regarding cancer-associated VTE, thromboprophylaxis is limited among outpatients, probably due to the sub-optimal predictive capacity of the existing tools used to predict the risk of experiencing a VTE event. The present work presents a new predictive score, the TiC-Onco score, which shows significantly better predictive power in this regard than the Khorana score.

The incidence of VTE in the present population at 6 months of follow-up was 18% (occurring in 71/391 patients); this is within the range of figures cited in a previous publication^24^ for the same follow-up period. It is also in agreement with the observation that the incidence of VTE is highest among patients with pancreatic cancer.^25^ However, no other clear differences between tumour types were seen with respect to the Khorana score, probably because a large proportion (46%) of the present patients had stage IV tumours.

The statistical analysis of the present data detected four genetic variants that were independently associated with VTE in outpatients with cancer (Table 2). These were combined into the algorithm for the Tic-Onco score, which initially allowed the patients to be classified as either at high or low risk of VTE. Among the patients in the TiC-Onco high risk group, 37% eventually suffered a VTE event, while 12% of those in the low risk group experienced the same (Table 4). However, when the three-tier Tic-Onco risk category system was contemplated (explained in Methods), 37% of the high risk, 18% of the moderate risk, and 6% of the low risk patients experienced a VTE event. In comparison, 22, 20, and 13% of the patients in the equivalent Khorana score categories experienced an event. The result obtained for the high risk Khorana score ≥3 (22%) is similar to that reported for high risk group at 6 months by other authors (18%) (*p *= 0.6).^26, 27^

The majority of genetic studies have excluded individuals with cancer-related thrombosis, and the relatively few studies that have been performed (which have mainly focused on the factor V Leiden and prothrombin G20210A genetic variants), have reported conflicting results.^1, 11, 12, 28–30^ These discrepancies are most likely due to the use of a single-marker, and inherent problems of low statistical power and poor reproducibility. The present work overcomes these problems by using several markers based on previous knowledge, a strategy that provided good results in previous work performed with non-oncology patients.^13^

Although the presently noted distribution of patients in the different Khorana risk categories is similar to that previously reported for the same follow-up time by other authors,^26, 27^ the predictive power of the TiC-Onco was found to be significantly greater than that of the Khorana Score. This superiority is demonstrated by a better AUC, better likelihood ratios, a higher PPV and NPV, and, importantly, a much higher sensitivity (49%).

In summary, this paper reports a clinical-genetic risk score that is significantly better than the Khorana score at identifying outpatients with cancer at high risk for experiencing a VTE event. Patients identified as being at high risk by the Tic-Onco risk score (using a specificity equal to that provided by the Khorana index as the cut-off) would likely benefit from thromboprophylaxis despite the risk of haemorrhage; they should, therefore, be seen as candidates for prophylactic treatment for VTE. The lower NNT of the Tic-Onco score reveals it can identify those patients most likely to benefit from prophylaxis. It is important that an accurate predictive tool like the TiC-Onco score be available if the morbidity associated with VTE is to be reduced, and because patients with cancer who experience a VTE are more likely to die than those who do not (31 vs. 11% in the present study; *p *< 0.001).

The reduced number of cancer types included might be seen as a weakness of the present study. However, it has the strengths of a multi-site setting, and a large proportion of patients in the advanced stages of tumour node metastasis classification.

As the risk of cancer-associated VTE is high even 6 months before cancer diagnosis, and the peak incidence is from 0 to 6 months^1, 31^ post-diagnosis, it is recommended that the TiC-Onco score be calculated at the moment cancer is suspected.^32, 33^
